# Probiotic Amelioration of Azotemia in 5/6th Nephrectomized Sprague-Dawley Rats

**DOI:** 10.1100/tsw.2005.86

**Published:** 2005-08-24

**Authors:** Natarajan Ranganathan, Beena Patel, Pari Ranganathan, Joseph Marczely, Rahul Dheer, Tushar Chordia, Stephen R. Dunn, Eli A. Friedman

**Affiliations:** ^1^ Kibow Biotech Inc., 3701 Market Street, Philadelphia, PA 19104, USA; ^2^ Department of Medicine, Division of Nephrology, Thomas Jefferson University, 1020 Locust Street, Room 353, Philadelphia, PA 19107-6799, USA; ^3^ Downstate Medical Center, State University of New York (SUNY) 450 Clarkson Avenue, Box 52, Brooklyn, NY 11203, USA

**Keywords:** uremia, probiotics, bacteriotherapy, chronic kidney disease, azotemia

## Abstract

The present study was to test the hypothesis that selected bacteria instilled into the gastrointestinal tract could help in converting nitrogenous wastes accumulated due to renal insufficiency into nontoxic compounds; thereby, ameliorating the biochemical imbalance. Herein we describe a prospective, blinded, placebo-controlled pilot study, using 5/6^th^ nephrectomized Sprague Dawley rat as a chronic renal failure model. The study group consisted of 36 nephrectomized and 7 non-nephrectomized (control) rats. After two-week nephrectomy stabilization, cohorts of six nephrectomized rats were fed casein-based diet plus one of the following regimens: (A) Control, (B) Placebo (casein-based diet without probiotics), (C) *Bacillus pasteurii*, (D) Sporolac®, (E) Kibow cocktail, (F) CHR Hansen Cocktail, and (G) ECONORM^TM^. Subsequently, blood (retro-orbital) and urine (collected for measurements of blood urea-nitrogen and creatinine respectively), body weight and bacterial counts (feces) were obtained at regular intervals. The study end-points were to determine if any of the probiotic dietary supplements facilitated, (1) decreased blood concentrations of uremic toxins, (2) altered renal function, and (3) prolonged survival. After 16 weeks of treatment, regimens C and D significantly prolonged the life span of uremic rats, in addition to showing a reduction in blood urea-nitrogen levels, concluding that supplementation of probiotic formulation to uremic rats slows the progression of azotemia, which may correlate with prolonged life span of uremic rats. Derivative trials of probiotic treatment of larger animals and humans will further assess the potential role of probiotic formulations in delaying the onset and clinical severity of clinical illness at different stages of renal failure.

### INTRODUCTION

Kidney dysfunction, often a consequence of other multisystem disorders such as diabetes, hypertension, and atherosclerosis, has a broad prevalence afflicting as many as 20 million Americans and 200 million world wide per year[1]. Consequent to progression from chronic kidney disease (CKD) to end-stage renal disease (ESRD), patients in "affluent" nations are treated with a kidney transplant or dialysis, most commonly hemo- or peritoneal dialysis. For patients with GFR (Glomerular Filtration Rate) 20-50 ml/min (estimated from the serum creatinine, concentration Cockcroft-Gault, or MDRD formula), termed as "pre-dialysis", only a few FDA-approved medicines are available, each targeting a single uremic solute such as potassium, phosphate, or water. Additionally, replacement of renal synthesized hormones including erythropoietin and vitamin D are increasingly employed to improve anemia and protect bones as renal failure worsens. The few alternatively available treatments for predialysis patients and the expensive hemodialysis procedure has stimulated research on alternative strategies, including use of probiotics, which might help to delay the need for dialysis and improvement of overall health. Additionally, the large population of individuals with lesser degrees of renal insufficiency might also benefit from a kidney "stabilizing" regimen.

Urea is the predominant nitrogen waste product of protein catabolism[2]. Although mechanisms involved in urea's toxicity are poorly understood[3], it is well established that urea contributes to the synthesis of other toxic moieties including guanidines and carbamylation products[4,5], or, at the very least, it appears to parallel the severity of renal dysfunction. Moreover, excess urea induces generation of free radicals and oxidative stress, which have been implicated in cellular damage[6]. Azotemia is a precondition to renal failure wherein nitrogenous waste products, chiefly urea, accumulate in the blood.

The concept of "enteric dialysis" as an alternative strategy for solute extraction in kidney failure is based on understanding that the intestinal mucosal surface functions as a semi-permeable membrane and is not a new concept. Driven by concentration gradient, solutes with elevated concentration in circulating blood diffuse from plasma into the lumen and a large portion of uremic solutes are differentially distributed throughout the bowel[7,8]. Uremic solutes might be extracted by binding to ingestible solute-specific sorbents within the gut[7], but this approach requires large quantities of each sorbent to be ingested daily. An innovative "enteric" approach to mitigate uremia using live bacteria that, when ingested, catabolize uremic solutes in the gut has been tested recently[9,10,11] and is further investigated in the present study.

We have evaluated numerous microbes for their ability to utilize various uremic solutes including urea, uric acid, creatinine, and most of the other potentially harmful biochemicals. By designing and formulating a regimen of probiotics targeting these substances in the bowel, we seek to improve the patient's overall health.

In the present study, a spore-forming urealytic gram-positive bacillus, lactic acid bacteria, and yeast were evaluated for bacteriotherapy of azotemia. In 5/6$^{th}$ nephrectomized rats we demonstrate that feeding with *Bacillus pasteurii* and *Lactobacillus sporogenes* attenuates blood urea levels measured as blood urea nitrogen (BUN) levels, which appears to correlate with a slowing of the progression of azotemia and increases the life span of uremic rats[12,13].

### MATERIALS AND METHODS

#### Microbial Cultures

- ***B. pasteurii* strain 6453 (Bp)** was obtained from American Type Culture Collection (ATCC). For preparation of spores, Bp was cultivated for 2–3 weeks on Tryptic Soy Agar (TSA) (Becton Dickinson and Company, Sparks, MD) pH 9.0 at 37°C, harvested, washed in sterile distilled water, resuspended in water, and pasteurized for 10–15 min in 80°C water bath. Spores that survived pasteurization were collected by centrifugation and viable spore count per gram of wet pellet was determined by most probable number (MPN) method in Tryptic Soy Broth pH 9.0. Pellets were stored at 4°C.

- **Sporolac®**: *L. sporogenes* probiotic strain was obtained from Sankyo Co. Ltd. (India). Viable cell count was determined by serial dilution method on MRS agar.
- **Kibow "Cocktail"** was prepared by lyophilizing commercial yogurt; 100-ml aliquots of yogurt were distributed in cryogenic sterile container and frozen at –70°C. Frozen aliquots were dried overnight in a Freeze Mobile 12 lyophilizer (Virtis Company, Gardiner, NY). Cell counts in initial culture, frozen samples, and freeze-dried formulation were determined by the standard plate count method on MRS (Man, Rogosa, and Sharpe) agar plates.
- **CHR Hansen Cocktail**: four freeze-dried probiotic strains (*L. delbrueckii* spp. *bulgaricus*, *L. acidophilus*, *Bifidus*, and *Streptococcus thermophilus)* were obtained from CHR Hansen BioSystems® (Milwaukee, WI). The strains were mixed in equal proportion by dry weight in a mini blender at low speed (4,000 rpm, three to four times for 30 sec at 1-min intervals). Viable cell count was determined by serial dilution method on MRS agar.
- **ECONORM™**: *Saccharomyces boulardii* strain was obtained from Dr. Reddy's Laboratories Ltd. (India). Viable cell count was determined by serial dilution method on Sabrouraud agar.

### Formulation Preparation

For the present study, food balls were prepared by moistening the casein-based diet (Dyets Inc, Bethlehem, PA; Lot No. 3036-2 Purified Rodent Diet 110900) with a single strain or mixture of strains, sterile 10% honey and milk mixture. The formulation was stored in a –70°C freezer until delivery to Thomas Jefferson University (TJU), Philadelphia. Aseptic conditions were maintained throughout the preparation. For formulation, composition, and dosage specifications, see Table 1.

**TABLE 1**
**Composition of Microbial Additive, Fed to Different Groups**

| Sr No. | Group | Microbial Additive | No. of Rats | Cfu/Dose*/Day** |
|---|---|---|---|---|
| 1 | (A) Control*** | No microbe(s) | 7 | |
| 2 | (B) Placebo | No microbe(s) | 6 | |
| 3 | (C) *Bacillus pasteurii* | *Bacillus pasteurii* | 6 | $1 \times 10^9$ cfu |
| 4 | (D) Sporolac® | *Lactobacillus sporogenes* | 6 | $1 \times 10^8$ cfu |
| 5 | (E) Kibow cocktail | *L. acidophilus*, *Streptococcus thermophilus*, *L. bulgaricus, Bifidus, L. casei, L. reuteri* | 6 | $1 \times 10^{10}$ cfu |
| 6 | (F) CHR Hansen Quarto | *L. acidophilus, L. bulgaricus, Bifidus, S. thermophilus* | 6 | $5 \times 10^{10}$ cfu |
| 7 | (G) (ECONORM™) | *Saccharomyces boulardii* | 6 | $1 \times 10^9$ cfu |

* Each dose correspondent to microbial count with casein diet food total of 5 g w/w.
** Each dose once a day.
*** Non-nephrectomized control.

### Study Design

A blinded pilot study, using a well-defined partially nephrectomized rat model of renal insufficiency, was conducted to evaluate *in vivo* application of probiotics for uremia therapy. Initially, 60 Sprague-Dawley rats with 5/6$^{th}$ two-step nephrectomies were performed at Charles River Labs, Inc. (Wilmington, MA). Two weeks after the two-stage surgery, rats were transported to TJU, where the study was conducted. Rats were individually caged and fed the casein-based diet, free from any microbes or yeast (Dyets Inc, Bethlehem, PA), in a temperature-controlled room. The study group consisted of 36 nephrectomized and 7 non-nephrectomized rats. After the nephrectomy stabilization period, cohorts of 6 rats were fed a casein-based diet plus one of the following regimen groups: (A) control, (B) placebo (casein-based diet without probiotics), (C) *B. pasteurii*, (D) Sporolac®, (E) Kibow cocktail, (F) CHR Hansen cocktail, and (G) ECONORM™. Groups C–G formed the treatment groups. Of the 36 nephrectomized rats, 18 males/18 females with the poorest renal function were ranked, selected, and placed into matching groups (B–G) of 6 each based on creatinine clearances, body weight (BW), and sex. The 7 non-nephrectomized rats (Group A) acted as nonuremic controls, while 6 nephrectomized rats (Group B) acted as uremic controls. Monthly BW, blood, and urine collections were obtained. Feces samples were collected before and after 8 weeks of feeding. This main diet (Dyets Lot No. 3036-2 Purified Rodent Diet 110900) was supplemented with additional daily feeding of five different microbial additives consisting of one of the labeled groups (Table 1). Supplements were hand fed to the rats daily (5 g/rat/day) for 16 weeks. The nonuremic control group (A) and the uremic control group (B) received the same food with no probiotic supplements.

Any deaths were noted within a maximum of 16 h. The TJU veterinarian or veterinary technician carefully examined all rats appearing ill or evidencing abnormal behavior. When a rat appeared in distress (for example, significant weight loss, ambulation problems, or failure to eat), the rat was humanely sacrificed by sedating with isofluorine before being put into the $CO_2$ chamber and the blood or tissue samples obtained. All rats were necropsied to ensure that the contralateral kidney had been removed. All procedures involving animals were performed according to the guidelines of the Institutional Animal Care and Use Committee[14].

### Sample Analysis

Blood urea-nitrogen concentration[15,16] was determined using standards and reagents supplied with Blood Urea-Nitrogen Reagent Kit (Cat. #535, Sigma, St. Louis, MO). Serum creatinine (Scr) concentrations were measured using direct end-point procedure with Creatinine Reagent sets from (Medicos, S. Plainfield, NJ) according to the manufacturer's instructions.

Limited analysis of fecal microbiota was performed for rats fed with regimen and placebo group. Fresh fecal samples were obtained from individual animals at baseline (before feeding) and after 8 weeks of feeding. Samples were placed into 5-ml Para-Pak Enteric Plus™ Transport System medium (Meridian Bioscience, Cincinnati, OH) and stored at –20°C for later analysis[17]. Fecal mass was resuspended in physiological peptone saline at an approximate concentration of 0.02 g/ml. Serial dilutions in saline were prepared and 100-μl aliquots from dilutions $10^{-5}$ and $10^{-7}$ were plated on MRS agar to obtain *Lactobacillus* spp. counts and TSB (pH 9.0) agar plates to enumerate the population of alkalophilic bacteria. Plates were incubated at 37°C for 48 h. MRS plates were kept in anaerobic BBL™ GasPak Pouches (Beckton Dickinson, Sparks, MD). The dilutions that had more than 50 colonies per plate were taken into account. Final counts are presented as log cfu/g of feces.

### Study End-Points

The study end-points were to determine if there are (1) reduction or stabilization of BUN, Scr, or increase in BW value compared to placebo values; (2) change in creatinine clearance; and (3) prolonged survival of rats. In addition, an increase of total *Lactobacillus* count in feces is considered to have beneficial effects.

### Statistical Analysis

An ANOVA (two-sided) test with the Bonferroni correction was used to measure between group variables. The Fisher's Exact (one-sided) test was used to compare survival with respect to diets. Statistical analyses of data were measured by the Student's t-test (Microsoft Excel). All results are expressed as mean ± standard deviation, values of $p < 0.05$ considered statistically significant.

## RESULTS

Rat models of CKD are widely used to evaluate the effect of the proposed treatment on kidney function. In this model, $5/6^{th}$ of kidney tissue is removed surgically leaving an animal with about 15% of kidney function on recovery from operation. In the present study, our model was employed to evaluate the possible use of probiotic mixtures in bacteriotherapy for azotemia. This model typically underachieves a significant decrease in renal function, probably due to subsequent hypertrophy of the remnant kidney; hence, more rats had to have the surgical procedure in order to get enough with marked renal insufficiency. Our experience is that only 60–75% reaches the desired renal insufficiency needed for a good CKD model. No significant baseline difference was detected in any of the measured variables between any of the experimental (nephrectomized) group. None of the control (A) rats (Scr = 0.2 ± 0.07, n = 7) died during the course of study. The ANOVA (two-sided) test with the Bonferroni correction showed no significant difference in Scr, BUN, and BW between groups at baseline.

Rats subjected to partial nephrectomy, as expected, developed renal insufficiency. As can be seen in Table 2, the BUN and Scr levels at baseline were significantly higher than those of the control group ($p < 0.05$ for BUN and $p < 0.001$ for Scr). In addition, the average BUN levels of the placebo group rose progressively over the 16-week period. Surviving rats in the placebo group had BUN levels 5–6.5 times higher ($p < 0.001$) than those in the control group and about 1.5 times higher ($p < 0.05$) than placebo baseline BUN level. This may indicate that azotemia in partially nephrectomized rats is progressing with time. Scr levels in the placebo group did not increase significantly (Table 2) over baseline, although this observation may be attributed to the fact that Scr levels were not measured at the end of the study (week 16) due to technical difficulties.

The ANOVA discerned no significant difference between placebo and treatment groups (C–G) with respect to baseline measurements. The number of days of survival was the primary variable. The most significant finding was that rats in the regimen groups C and D appeared to sustain better survival. Group A rats exhibited 100% survival (summarized data in Table 3).

A significant difference between the regimen groups with respect to mean survival days was detected ($p = 0.0375$; one-sided Fisher's test) as is seen that Group C and D appeared to be more effective than B ($p = 0.042$), G ($p = 0.041$), and F ($p = 0.035$). Group G had the highest morbidity and mortality, in addition to showing the highest percent increase in Scr and BUN levels, and decreases in creatinine clearance (see Table 4). Group G also displayed second-worst BW loss, confirming that this diet supplement was ineffective. Group B showed a significant increase in Scr and BUN levels as well as decrease in BW, which are typical symptoms at the CKD stage of kidney failure, from low-nephron condition, though the change in creatinine clearance was not found to be significant. Group F showed high mortality. Group E seemed effective with moderate mortality and morbidity, while showing minimal change with BUN and Scr levels. Group C showed increase in BW and decrease in BUN. Overall, Group C and D were found to be superior compared to other groups.

Limited analysis of fecal microbiota in rats fed with placebo and probiotics revealed no effect on *Lactobacillus* spp. in the rat intestine by all regimen groups, except for group D. It showed significant increase in total *Lactobacillus* count from baseline ($p < 0.01$) (see Table 5). To evaluate possible proliferation of Bp in the uremic intestine, we also enumerated the population of alkalophilic bacteria in the feces obtained from both placebo- and Bp-fed rats (Group C). No significant differences in counts of alkalophilic bacteria were observed after 8 weeks of daily feeding with $10^9$ cfu of Bp (log cfu/g 9.50 ±

**TABLE 2**
**Comparison of Survival, BW, BUN, and Scr Concentrations of Control Rats and Placebo[1]**

| Group | Period | Survival (No. of Rats)(M/F) | BW (g) | BUN (mg/dl) | Scr (mg/dl) |
|---|---|---|---|---|---|
| Control (A) | Baseline | 7 (4/3)[2] | 305.7 ± 14.8 | 12.0 ± 0.7 | 0.20 ± 0.03 |
| | 4 weeks | 7 | 458.0 ± 45.5 | 13.0 ± 2.2 | 0.20 ± 0.03 |
| | 8 weeks | 7 | 541.0 ± 63.0 | 15.0 ± 0.8 | 0.40 ± 0.04 |
| | 16 weeks | 7 | 558.0 ± 67.8 | 16.0 ± 3.3 | Not determined |
| Placebo (B) | Baseline | 6 (3/3)[2] | 265.5 ± 14.5 | 65.2 ± 6.9* | 1.20 ± 0.20** |
| | 4 weeks | 6 (3/3)[2] | 326.5 ± 28.7 | 55.5 ± 12.0* | 0.98 ± 0.19** |
| | 8 weeks | 4 (2/2)[2] | 374.8 ± 48.5 | 99.8 ± 30.2* | 1.48 ± 0.36** |
| | 16 weeks[3] | 2 (1/1)[2] | 436.0 ± 80.0* | 104.0 ± 10.0**,*** | Not determined |

1 Data are mean ± SEM for the surviving animals.
2 Numbers of surviving males/females.
3 Mean ± SD.
* Significantly different from control group, $p < 0.05$.
** Significantly different from control group, $p < 0.01$.
*** Significantly different from baseline, $p < 0.05$.

**TABLE 3**
**Survival of Rats in Days of Placebo Group (B) and the Treatment Groups (C–G)**

| Group | Alive | Dead | % Survival | Mean Survival (Days) | Standard Deviation |
|---|---|---|---|---|---|
| B | 2 | 4 | 33.3 | 115.8 | 39.3 |
| C* | 4 | 2 | 66.7 | 148.5 | 14.1 |
| D* | 5 | 1 | 83.3 | 149.3 | 16.3 |
| E | 3 | 3 | 50.0 | 126.5 | 32.9 |
| F | 2 | 4 | 33.3 | 116.2 | 35.7 |
| G | 2 | 4 | 33.3 | 111.2 | 44.0 |

* $p < 0.05$ from G, B, F, E (Fishers test), and no deaths appeared in the non-nephrectomized Group A.

**TABLE 4**
**Percent Change in Clinical Parameters (Scr, BUN, CrCl, BW) from Baseline to Death/Sacrifice***

| Group | % Δ Scr | % Δ BUN | % Δ CrCl | % Δ Body Wt. |
|---|---|---|---|---|
| B | 89.0 ±36.5 (n = 4) | 122 ± 7.1 (n = 3) | 0.60 ± 51.4 (n = 2) | –3.4 ± 9.0** (n = 6) |
| C | 33.9 ± 38.1 (n = 6) | –10.9 ± 37.2** (n = 6) | –14.9 ± 32.1** (n = 5) | 24.6 ± 4.4 (n = 6) |
| D | 14.8 ± 15.2 (n = 6) | 39.7 ± 27.6 (n = 6) | –29.9 ±16.5** (n = 5) | 21.1 ±13.1 (n = 6) |
| E | 12.5 ± 17.8 (n = 6) | 47.0 ± 38.8 (n = 5) | 39.1 ± 53.1 (n = 4) | 8.7 ± 12.7 (n = 6) |
| F | 154 ± 71.8 (n = 6) | 83.7 ± 60.3 (n = 5) | 120 ± 156 (n = 2) | –0.20 ± 12.1** (n = 6) |
| G | 182 ± 124 (n = 6) | 116 ± 87.9 (n = 3) | –40.0 ± 18.1** (n = 2) | –1.9 ±7.9** (n = 6) |

* Data are means and SEM of n samples. ( ) Values in parentheses indicates number of rats at the end of study (death/sacrifice).
** Indicates decrease in value.

**TABLE 5**
**Effect of Probiotic Formulation on *Lactobacillus spp.* and *Alkalophilic* spp. Before and After 8 Weeks of Treatment**

| Group | Microbial Supplement | Period | Count Log cfu/g of Feces | Times Increase from Baseline |
|---|---|---|---|---|
| B (Placebo) | *Lactobacillus* spp. | Baseline | 8.76 ± 0.40 | |
| | | 8 weeks | 8.66 ± 0.34 | 0.988 |
| | *Alkalophilic* spp. | Baseline | 8.46 ± 0.54 | |
| | | 8 weeks | 8.00 ± 0.00 | 0.945 |
| C | *Lactobacillus* spp. | Baseline | 8.23 ± 0.97 | |
| | | 8 weeks | 8.36 ± 0.86 | 1.016 |
| | *Alkalophilic* spp. | Baseline | 9.30 ± 0.39 | |
| | | 8 weeks | 9.50 ± 0.26 | 1.021 |
| D | *Lactobacillus* spp. | Baseline | 9.00 ± 0.24 | |
| | | 8 weeks | 9.31 ± 0.07* | 1.035 |
| E | *Lactobacillus* spp. | Baseline | 8.87 ± 1.26 | |
| | | 8 weeks | 8.79 ± 1.16 | 0.991 |
| F | *Lactobacillus* spp. | Baseline | 8.41 ± 1.12 | |
| | | 8 weeks | 9.23 ± 0.58 | 1.097 |
| G | *Lactobacillus* spp. | Baseline | N/D | |
| | | 8 weeks | N/D | N/D |

Data are mean ± SD. * $p < 0.01$ from placebo.

0.26) as compared to baseline (log cfu/g 9.30 ± 0.39), suggesting that Bp does not proliferate in the intestine. Group G, fed with *S. boulardii,* feces analysis was not performed due to technical difficulty.

### DISCUSSION

Current therapies to remove uremic solutes in patients afflicted with renal insufficiency (CKD) or ESRD include peritoneal dialysis, hemodialysis, and kidney transplantation. Each of these expensive and time-consuming procedures is associated with high morbidity and is only available in industrialized countries. In underdeveloped countries, uremia is generally untreated, which usually leads to death. Predialysis patients may be treated with protein and potassium diet restrictions, erythropoietin, and to some extent compound-specific interventions such as angiotensin converting enzyme (ACE) inhibitors or angiotensin receptor blockers (ARB), and Renagel® to reduce hyperphosphatemia or Kaexylate® to extract retained potassium are the only few applicable therapies to slow the progression of kidney disease available. In recent years, efforts have been undertaken to mitigate uremia in animals and humans by administration of live cultures of naturally existing[9] or genetically engineered microbes[10,18]. Earlier studies by Simenhoff et al. and a more recent, yet unpublished, double-blind, placebo-controlled trial with 30 chronic dialysis patients (compared placebo to *L. acidophilus*), demonstrated that the 15 hemodialysis patients fed *L. acidophilus* (NCFM™) had significantly lower blood dimethylamine and nitrodimethylamine levels[19,20], two uremic toxins with origin in the small bowel[21]. Taken together, these studies not only prove the concept of *in situ* continuous "enteric dialysis", but also give hope to the millions of people with kidney dysfunction of diverse etiology. The present study is the culmination of extensive *in vitro* preliminary experimentation to devise a probiotic regimen for bacteriotherapy in uremia. Current results are strongly supported by *in vivo* studies documenting beneficial effects of probiotic feeding in laboratory models of renal insufficiency. Supplementation of probiotics for 16 weeks attenuated BUN levels in 5/6$^{th}$ nephrectomized rats, slowed the progression of azotemia observed in the placebo group, and prolonged the life of uremic rats. However, the most notable observations are Group C and D, which stand apart from the others showing significantly less mortality ($p < 0.05$) and highest BW gain.

### CONCLUSION

This pilot study found that probiotic dietary supplements used in Groups C and D regimen enhanced survival of 5/6$^{th}$ nephrectomized rats while slowing the progress of renal injury induced by reduced nephron mass. Our results support the view that dietary supplements will proffer beneficial application for uremia either as a food, a food supplement, or as pharmaceutical preparation. Derivative trials of probiotic treatment of larger animals and humans will further assess the potential role of probiotic formulations in delaying the onset and clinical severity of clinical illness at different stages of renal failure. One could speculate that a standard renoprotective treatment, if instituted early and including a probiotic arm directed at arresting the pathophysiology that accompanies the early uremic syndrome, might alter the course of renal disease delaying the need for dialysis.

The study supports our intent to conduct the derivative trials (employing bigger animals and greater numbers of subjects) using a single or possibly two of the dietary supplements with measurement of additional nitrogenous wastes such as guanidine, indoxylsulphate, creatinine, and possibly an enzyme, perhaps Glutathione Transferases (GST) for uremia.

**Limitation:** low numbers of rats, fecal sample collection.

### ACKNOWLEDGMENTS

This research was supported in part by the National Institute of Diabetes and Digestive Diseases (NIH) SBIR Grant Award #1R44 DK55403.
